# Postnatal pelvic floor muscle stiffness measured by vaginal elastometry in women with obstetric anal sphincter injury: a pilot study

**DOI:** 10.1007/s00192-019-04136-z

**Published:** 2019-12-04

**Authors:** Dilly O. C. Anumba, Siobhán Gillespie, Swati Jha, Shahram Abdi, Jenny Kruger, Andrew Taberner, Poul M. F. Nielsen, Xinshan Li

**Affiliations:** 1 Academic Unit of Reproductive and Developmental Medicine, Faculty of Medicine Dentistry and Health, The University of Sheffield, Jessop Wing, Tree Root Walk, Sheffield, S10 2SF UK; 2 Insigneo Institute for in silico Medicine, The University of Sheffield, Sheffield, UK; 3 Clinical Radiology, Sheffield Teaching Hospitals, Sheffield, UK; 4 Auckland Bioengineering Institute, University of Auckland, Auckland, New Zealand; 5 Department of Engineering Science, University of Auckland, Auckland, New Zealand; 6 Department of Mechanical Engineering, The University of Sheffield, Sheffield, UK

**Keywords:** Pregnancy, Urogynaecology, High-risk pregnancy, Delivery, Incontinence, Perineum

## Abstract

**Introduction and hypothesis:**

Vaginal childbirth is associated with pelvic floor muscle (PFM) damage in a third of women. The biomechanics prediction, detection and management of PFM damage remain poorly understood. We sought in this pilot study to determine whether quantifying PFM stiffness postnatally by vaginal elastometry, in women attending a perineal trauma clinic (PTC) within 6 months of obstetric anal sphincter injury, correlates with their antecedent labour characteristics, pelvic floor muscle damage, or urinary/bowel/sexual symptoms, to inform future definitive prospective studies.

**Methods:**

In this pilot study, we measured postnatal PFM stiffness by vaginal elastometry in 54 women. A subset of participants (*n* = 14) underwent magnetic resonance imaging (MRI) to define any levator ani (LA) muscle defects from vaginal childbirth. We investigated the association of PFM stiffness with demographics, labour and delivery characteristics, clinical features and MRI evidence of LA damage.

**Results:**

Raised maternal BMI was associated with reduced pelvic floor stiffness (r = −0.4; *p* < 0.01). Higher stiffness values were associated with forceps delivery for delayed second stage of labour (*n* = 14) vs non-forceps vaginal delivery (*n* = 40; 630 ± 40 N/m vs 500 ± 30 N/m; *p* < 0.05), and a non-significant trend towards longer duration of the second stage of labour. Women with urinary, bowel or sexual symptoms (*n* = 37) demonstrated higher pelvic floor stiffness values than those without (570 ± 30 N/m vs 450 ± 40 N/m; *p* < 0.05).

**Conclusions:**

A history of delayed second stage of labour and forceps delivery was associated with higher PFM stiffness values in the postnatal period. Whether high pelvic muscle stiffness antenatally is a risk factor for instrumental vaginal delivery and LA avulsion is unknown.

## Introduction

Vaginal childbirth causes perineal muscle [1], and pelvic floor (mainly the levator ani, LA) muscle damage, contributing to short-term and long-term urinary incontinence and pelvic organ prolapse [2]. Perineal trauma is usually evident on clinical examination immediately after childbirth when surgical repair may be undertaken if required. Occasionally, anal sphincter damage may not be detected until the woman complains of fecal or urinary urgency/incontinence several days to weeks after childbirth, when trans-labial or trans-anal sonography may identify a defect. In contrast, damage to the LA muscle resulting from vaginal delivery is usually initially undetected because many women are asymptomatic and investigating such injury is not routine [3]. Such unrecognized damage may be identified later, for instance, when menopause and aging associated with low oestrogen lead to clinical presentation with pelvic organ prolapse (POP) and/or urinary incontinence (UI) [4]. The latter conditions affect more than 20% of women [5] and significantly diminish quality of life [6].

The aetiology of, and predisposing factors to, LA muscle damage during childbirth have remained unclear, but the potential contributions of vaginal delivery, the use of forceps, anal sphincter rupture, episiotomy, epidural anaesthesia and oxytocin use [7, 8] have been described. It has been postulated that pelvic floor muscle (PFM) tone or stiffness may influence the duration and course of the second stage of labour, and consequently the propensity of the PFM to injury in a subsequent pregnancy. However, no studies have investigated the impact of PFM tone or stiffness on the second stage of labour or LA muscle avulsion injuries. Although computational modelling has provided some insight into the biomechanics of the pelvic floor during parturition [9, 10], it is not clear what factors affect the susceptibility of the LA muscle to injury and how their mitigation can prevent such injury.

In recent years, pelvic floor dynamometry or elastometry (vaginal elastometry), has emerged as a potential tool for the functional evaluation of pelvic floor mechanics [11, 12]. In this pilot study we investigated, in the setting of a perineal trauma clinic (PTC), whether PFM stiffness measured by elastometry in the postnatal period correlates with the duration of the second stage of labour and the requirement for instrumental (forceps) delivery for delayed second stage of labour (defined in this study as a second stage lasting longer than 120 min) in the antecedent pregnancy. In a subset of patients, we also explored whether there was any relationship between LA muscle defects on pelvic magnetic resonance imaging (MRI) and PFM stiffness measured by vaginal elastometry.

## Materials and methods

In this observational pilot study, we employed a hand-held vaginal elastometer to measure postnatal PFM stiffness in 54 consenting attendees of a postnatal PTC at a tertiary teaching hospital in the UK (the Jessop Wing of the Royal Hallamshire Hospital Sheffield) between August 2014 and October 2015. Their obstetric history and diagnosis were retrospectively cross-checked from the records—all of the women had had at least one previous vaginal delivery and suffered an obstetric anal sphincter injury (OASI) in their most recent pregnancy. A subset of participants (*n* = 14) had undergone MRI scans to diagnose the presence of any LA muscle defects resulting from vaginal childbirth. We investigated the association of PFM stiffness values (the primary outcome) with patient demographics, labour characteristics, mode of vaginal delivery, clinical features, and evidence of LA damage on MRI.

### The setting

All patients who sustain a third (3A, B or C) or fourth degree OASI in their last pregnancy are reviewed between 3 and 6 months postnatally at the PTC, as per guidelines recommended by the Royal College of Obstetricians and Gynaecologists (RCOG) [13]. At each clinic visit, the patients completed a symptom questionnaire, assessing all aspects of pelvic floor function in the domains of urinary, bowel, vaginal and sexual function using a validated questionnaire—the electronic Personal Assessment Questionnaire-Pelvic Floor (ePAQ-PF) [14]. They are clinically evaluated, including a perineal examination for defects, scars, evidence of uterovaginal descent or anal sphincter defects. Pelvic floor muscle contraction strength is quantified using the Oxford score [15]. Patients who have either fecal urgency or incontinence, a defect on clinical examination of the anal sphincter or a major OASI (third- or fourth-degree tear) are investigated further by endo-anal physiology, endoanal ultrasound and pudendal nerve latency studies. Patients with symptoms of fecal urgency and incontinence are initially offered conservative management using biofeedback.

### The participants

**Fig. 1:**
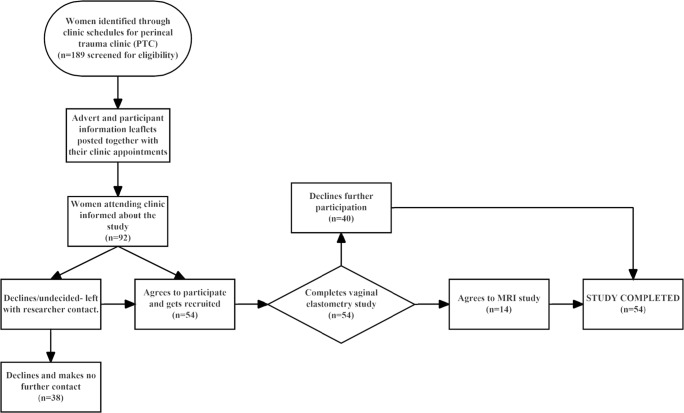
Flow chart depicting participant recruitment and investigation during the study

Figure 1 is a flow chart that summarizes participant recruitment and investigation during the study. Participants were recruited from the cohort of patients referred to the PTC. All women referred to the PTC were eligible for inclusion in the study if they agreed. However, women who had a history of previous perineal surgery, or surgery for genital prolapse or stress UI, were excluded from the study. At the time of sending out an invitation/appointment letter to attend the clinic, prospective participants were also sent information leaflets explaining the study and inviting them to consider participating. When they attended the clinic, the clinician researchers ascertained whether they wished to take part and provided them with detailed information about the study. Women consenting to participate gave informed written consent and were assessed at the end of the clinical consultation, on the same day or subsequently.

### Vaginal elastometry

We employed a portable vaginal elastometer, developed at the Auckland Bioengineering Institute, to quantify passive stiffness of the LA muscles [16]. It is a hand-held automated instrumented speculum that consists of a hand-piece comprising two aluminium arms, with detachable acetyl plastic speculum ends, actuated via a load cell. The tip of the speculum is wider than the neck to focus the measurements at the level of the puborectalis portion of the LA muscle group by reducing contributions from perineal muscles. The hand-piece is connected to a control box with a data acquisition device that communicates with a computer via a USB connection. The device measures the passive force and the displacement (i.e. speculum separation), and displays the data in a graph. Stiffness values are quantified in Newton/m (N/m). The portable device prototype is highly acceptable, consistent and repeatable in both non-pregnant and pregnant women [17].

The measurements were carried out by a single trained operator—the research nurse/midwife—employing a predefined protocol as previously described [17]. Briefly, the patient was first instructed to perform a maximum voluntary contraction of her perineal muscles whilst the PFM was palpated clinically to determine the optimal placement of the speculum at the level of the PFM. Following insertion of the speculum in the closed position, the device was opened in 10-mm stepwise increments to a maximum tolerated aperture of 40 mm (which demonstrated high reliability in preliminary studies), slightly lower than previously reported [17] as some women with OASI could not tolerate the 50-mm aperture. At each step, force measurements were acquired over 1 s after a 3-s relaxation time. The measured force and displacement data sets were recorded at a frequency of 100 samples per second. The procedure was carried out three times: the first cycle was to allow for tissue preconditioning, and to familiarize the patient with the measurement procedure whilst the definitive measurements were taken over the subsequent two cycles. The force–displacement curve was used to calculate passive stiffness (*k*) from approximately the most linear portion of the force–displacement curve, which was an aperture of between 35 mm and 40 mm for all women. Averaged force and displacement measurements from the two measurement cycles were used in the analysis.

A pelvic MRI scan was subsequently conducted within 2 weeks of the assessment on 14 consenting symptomatic (*n* = 8) and non-symptomatic (*n* = 6) women, as the “gold standard” for defining pelvic floor muscle damage by showing LA muscle defects [18]. The images were acquired on a 3.0-Tesla MRI scanner (Ingenia; Philips Medical Systems, Best, The Netherlands). T2-weighted scans were obtained in sagittal, axial and coronal planes in supine position. The slice thickness was 3 mm with a 0.3-mm gap and a 1-mm gap in the sagittal plane. T2-weighted volume isotropic turbo spin echo acquisition (VISTA) scans, 1-mm slice thickness with no gap and reformat in the coronal plane were also acquired. The images were reviewed by one examiner (SA) with reference to the previously published data on the appearance of LA muscle abnormalities [19].

### Statistical analysis

Statistical analysis was carried out employing the software MedCalc® version 17.6 (Belgium). Descriptive statistics were employed to summarize patient demographics, clinical features and measurements, in addition to pelvic stiffness measurements on vaginal elastometry. The normality of data distribution was ascertained by the D’Agostino–Pearson test. Parametric (Student’s *t* tests) and non-parametric (Mann–Whitney *U*) tests were used as appropriate. The relative association between postnatal PFM stiffness and prolonged second stage of labour (defined as the duration between full cervical dilatation and complete delivery of the baby), forceps delivery in the antecedent pregnancy, and presentation with urinary, bowel or sexual symptoms was also compared by estimating the area under the receiver operating characteristic (ROC) plots of sensitivity against specificity. Fisher’s exact test was used to determine associations between the categorical variables symptoms of pelvic floor/perineal trauma and MRI evidence of LA muscle defects/avulsion [20].

### Ethical approval

The study was approved by the North Sheffield Research Ethics committee (NRES REC Number 14/NE/1014).

## Results

Table 1 summarizes the demographic features and index clinical birth outcomes of study participants.

**Table 1:** Subject details and index birth outcomes

	Mean	SD	Median	Minimum	Maximum
Age (years)	30.3	4.7	31.0	21.0	40.0
BMI (kg/m^2^)	26.4	5.7	25.7	18.4	44.3
Weight (kg)	71.0	16.9	68.1	47.0	118.1
Height (cm)	164.9	6.7	164.0	148.0	183.0
Baby weight (kg)	3.7	0.5	3.7	2.7	5.1
Gestation (days)	281.7	7.7	281.0	261.0	295.0
Pelvic floor muscle stiffness (N/m)	530	190	520	140	950
Oxford Score	2.5	1.2	3.0	0.0	5.0
Length of second stage (min)	78.9	64.6	60.5	8.0	300.0

**Fig. 2:**
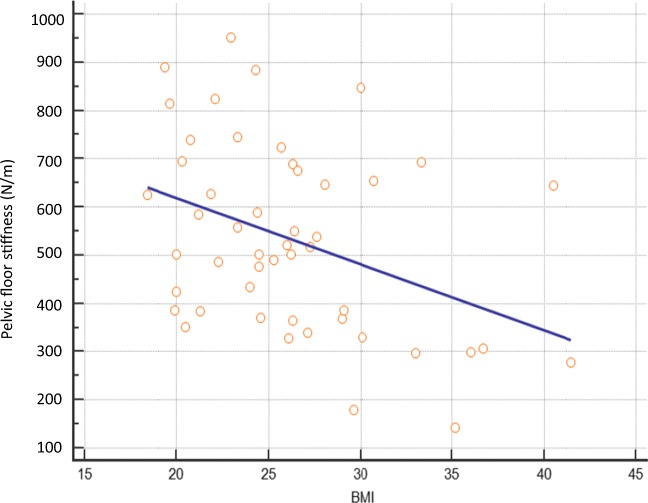
Scatter diagram showing a negative correlation between maternal body mass index (BMI) and pelvic floor muscle stiffness (r = −0.4; *p* < 0.01)

Of the 54 participants, prior to the index pregnancy associated with OASI, 40 (74%) had been nulliparous before, whereas 14 (27%) had delivered one previous child. Of the parous women 7, 5 and 2 had had spontaneous vaginal, instrumental vaginal, or caesarean delivery respectively. Sixteen (29%) were current smokers, whereas 39 (71%) were not. Raised maternal BMI, but not maternal age or smoking status, was associated with reduced PFM stiffness (r = −0.4; *p* < 0.01; Fig. 2). Oxford Scores of pelvic muscle tone did not correlate with PFM stiffness scores (*r* = 0.06, *p* = 0.68).

Tables 2, 3, 4, 5, and 6 shows the mean PFM stiffness measurements of study cohorts categorised by obstetric factors described to be associated with LA muscle damage, including the duration of the second stage of labour (defined as being from full cervical dilatation to complete fetal delivery), the method of vaginal delivery (spontaneous vaginal delivery, ventouse, forceps) [21] and the presence of any urinary (urgency or stress incontinence), bowel (fecal or flatus incontinence or urgency), or sexual (dyspareunia) symptoms. Stiffness values are also shown for the subset of participants (*n* = 14) who had MRI evidence of LA muscle avulsion/defects compared with those who had none.

**Table 2:** Pelvic floor muscle stiffness measurements categorised by duration of the second stage of labour

	<2 h		≥2 h		
Assessed parameter	*n*	Mean (SE)	*n*	Mean (SE)	*p* value
Pelvic muscle stiffness (N/m)	37	520 (30)	11	590 (40)	0.19
Oxford Score	39	2.54 (0.18)	12	2.29 (0.41)	0.54

**Table 3:** Pelvic floor muscle stiffness measurements categorised by mode of vaginal delivery: forceps or non-forceps

	Non-forceps delivery		Forceps delivery		
Assessed parameter	*n*	Mean (SE)	*n*	Mean (SE)	*p* value
Pelvic muscle stiffness (N/m)	40	500 (30)	14	630 (40)	<0.05
Oxford Score	40	2.60 (0.19)	14	2.08 (0.37)	0.17
Duration of the second stage of labour (min)	40	65 (9)	14	117 (17)	<0.01

**Table 4:** Pelvic floor muscle stiffness measurements categorised by mode of vaginal delivery: spontaneous vaginal (SVD) or instrumental (ventouse and forceps) delivery

	SVD		Instrumental		
Assessed parameter	*n*	Mean (SE)	*n*	Mean (SE)	*p* value
Pelvic muscle stiffness (N/m)	37	490 (40)	17	620 (40)	<0.05
Oxford Score	37	2.66 (0.20)	17	2.09 (0.32)	0.13
Duration of the second stage of labour (min)	37	53 (6.6)	17	131 (17.5)	<0.01

**Table 5:** Pelvic floor muscle stiffness measurements categorised by presence of any urinary, bowel or sexual symptoms

	No		Yes		
Assessed parameter	*n*	Mean (SE)	*n*	Mean (SE)	*p* value
Pelvic muscle stiffness (N/m)	17	450 (40)	37	570 (30)	<0.05
Oxford Score	17	2.03 (0.27)	37	2.69 (0.20)	0.06
Duration of the second stage of labour (min)	17	76 (14)	37	80 (11)	0.85

**Table 6:** Pelvic floor muscle stiffness measurements categorised by MRI evidence of an LA defect

	No		Yes		
Assessed parameter	*n*	Mean (SE)	*n*	Mean (SE)	*p* value
Pelvic muscle stiffness (N/m)	6	460 (80)	8	540 (50)	0.25
Oxford Score	6	2.33 (0.42)	8	2.14 (0.46)	0.76
Duration of the second stage of labour (min)	6	73.5 (22)	8	102.4 (25)	0.40

**Fig. 3:**
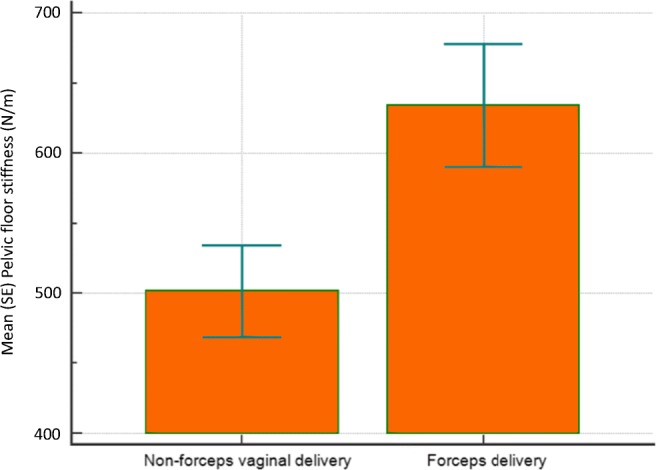
Histogram illustrating higher mean (SE) pelvic floor muscle stiffness in women who had forceps delivery for a prolonged second stage of labour (630 ± 40 N/m) compared with women who had a non-forceps vaginal delivery (510 ± 40 N/m; *p* < 0.05)

Higher PFM stiffness was associated with instrumental vaginal delivery versus spontaneous-delivered women (620 ± 40 N/m vs 500 ± 40 N/m respectively; *p* < 0.05), particularly forceps delivery for delayed second stage of labour versus non-forceps vaginal delivery (630 ± 40 N/m vs 510 ± 40 N/m respectively; *p* < 0.05; Fig. 3). A trend towards higher PFM stiffness values in women who had a prolonged second stage of labour (defined here as >2 h) did not attain statistical significance (*p* = 0.19). A positive correlation trend between labour duration and pelvic stiffness values did not attain statistical significance (r = 0.2, *p* = 0.18). All the women who had evidence of unilateral or bilateral LA avulsion injury, consistent with previous trauma from vaginal childbirth, had urinary, bowel or sexual symptoms compared with 2 of the 6 women who had no MRI features of LA muscle defects (Chi-squared statistic 6.93; df = 1; *p* < 0.01). Furthermore, women with MRI evidence of avulsion demonstrated non-significantly higher PFM stiffness values than women with intact LA muscle. Women who presented with symptoms also demonstrated higher pelvic stiffness values than asymptomatic women (570 ± 30 N/m vs 450 ± 40 N/m; *p* < 0.05). A subset of study participants who were primiparae at the time of the study showed similar trends, but mean PFM stiffness was only significantly higher in women presenting with urinary, bowel or sexual symptoms (Tables 7, 8, 9, 10, and 11).

**Table 7:** Pelvic floor muscle stiffness measurements in the sub-group with no previous delivery history, categorised by duration of second stage of labour

	<2 h		≥2 h		
Assessed parameter	*n*	Mean (SE)	*n*	Mean (SE)	*p* value
Pelvic muscle stiffness (N/m)	20	548 (36)	11	587 (36)	0.59

**Table 8:** Pelvic floor muscle stiffness measurements in the sub-group with no previous delivery history categorised by mode of vaginal delivery: forceps or non-forceps

	Non-forceps delivery		Forceps delivery		
Assessed parameter	*n*	Mean (SE)	*n*	Mean (SE)	*p* value
Pelvic muscle stiffness (N/m)	20	549 (44)	10	598 (40)	0.48

**Table 9:** Pelvic floor muscle stiffness measurements in the sub-group with no previous delivery history: spontaneous vaginal (SVD) or instrumental (ventouse and forceps) delivery

	SVD		Instrumental		
Assessed parameter	*n*	Mean (SE)	*n*	Mean (SE)	*p* value
Pelvic muscle stiffness (N/m)	18	536 (40)	13	598 (40)	0.37

**Table 10:** Pelvic floor muscle stiffness measurements in the sub-group with no previous delivery history categorised by presence of any urinary, bowel or sexual symptoms

	No		Yes		
Assessed parameter	*n*	Mean (SE)	*n*	Mean (SE)	*p* value
Pelvic muscle stiffness (N/m)	11	468 (49)	21	614 (38)	<0.05

**Table 11:** Pelvic floor muscle stiffness measurements in the sub-group with no previous delivery history categorised by MRI evidence of an LA defect

	No		Yes		
Assessed parameter	*n*	Mean (SE)	*n*	Mean (SE)	*p* value
Pelvic muscle stiffness (N/m)	5	450 (63)	5	590 (49)	0.12

**Fig. 4:**
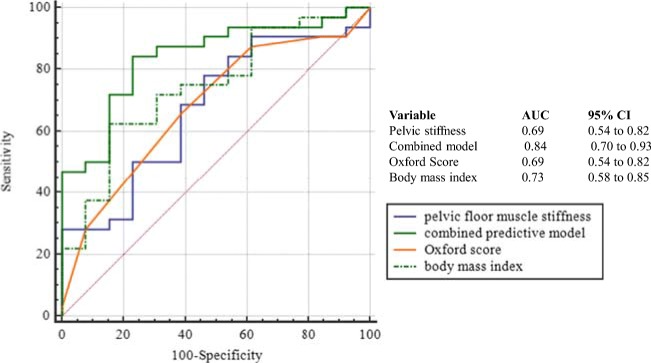
Relative predictive association of BMI, PFM stiffness and the Oxford Score with any symptoms of perineal/pelvic floor injury, expressed as the area under the receiver operating characteristic curves (AUC) of sensitivity against specificity

Figure 4 depicts the relative association of BMI, PFM stiffness and the Oxford grading score with any symptoms of perineal/pelvic floor injury, expressed as the area under the ROC curves (AUCs) of sensitivity against specificity. All three parameters were associated with presentation to the PTC with symptoms, the BMI (AUC 0.73) more so than PFM stiffness and the Oxford Score (AUCs both 0.69). Furthermore, combining BMI, PFM stiffness and the Oxford Score improved the association with presentation with urinary or bowel or sexual symptoms (AUC 0.84; 95% CI 0.70, 0.93; *p* < 0.0001, sensitivity 85%, specificity 79%, positive predictive value (PPV) 90, negative predictive value (NPV) 69, positive likelihood ratio (+ve LR) 4.0, and negative likelihood ratio (−ve LR) 0.2; Fig. 4) compared with any of the parameters alone. However, only BMI (*b* −0.18, SE 0.08, Wald statistic 4.9; *p* = 0.03) and Oxford Scores (*b* 0.83, SE 0.33, Wald statistic 6.72; *p* = 0.01) at the time of assessment was independently associated with ongoing urinary, bowel or sexual symptoms.

## Discussion

We describe, for the first time, postnatal PFM stiffness measured using a portable vaginal elastometer in a cohort of women attending a postnatal PTC who had experienced major OASI during vaginal childbirth. We note that the maternal BMI correlated negatively with PFM stiffness, and that PFM stiffness is higher in women who required instrumental (forceps), rather than spontaneous, vaginal delivery for delayed second stage of labour, defined for the purpose of this study as a second stage lasting longer than 120 min. Our small sub-cohort of women who demonstrated LA muscle defects on pelvic MRI assessment, on average, experienced longer second stages of labour and higher passive stiffness values than women without muscle defects, but these did not attain statistical significance. All the women with LA muscle defects on MRI (8/8) presented with persisting symptoms whereas only 2 of the 6 women with intact LA muscle appearances had bowel, urinary or sexual symptoms.

Our observation that high postnatal PFM stiffness values showed a trend towards association with delayed second stage of labour, instrumental (forceps) vaginal delivery and LA muscle defects on MRI is intriguing given that it could be expected that childbirth-induced trauma to the LA muscle, especially when attributable to instrumental forceps delivery for prolonged second stage of labour, would result in lower postnatal LA muscle passive stiffness values, and a higher likelihood of postnatal persistence of urinary, bowel, or sexual symptoms, although these symptoms may be attributable to perineal muscle rather than levator trauma. However, it is plausible that higher PFM stiffness antenatally is a risk factor for prolonged second stage of labour, instrumental vaginal delivery, and/or LA muscle avulsion injuries, and persists postnatally. Indeed, a recent pilot study that assessed LA muscle avulsion injuries by perineal ultrasonography noted significant differences between antenatal and postnatal PFM stiffness measurements, but no significant differences in antenatal stiffness between women who sustained LA avulsion injuries and those who did not [17]. However, this study reported that a noted rise in PFM stiffness values between antenatal and postnatal assessments was significantly lower in the avulsion group. Consistent with our observations in this predominantly Caucasian population, this study also reported more avulsion injuries in their European cohort, which demonstrated higher PFM stiffness values than the Polynesian cohort, also suggesting that high antenatal LA muscle passive stiffness may be a risk factor for avulsion injuries.

Limited sample sizes, differing imaging approaches, and diagnostic criteria for obstetric LA muscle avulsion [22] preclude definite conclusions to be drawn regarding the potential value of antenatal PFM stiffness measurements by elastometry for predicting LA muscle trauma. It may be speculated that the latter might be specifically assessed using approaches that combine elastography and MRI/ultrasound. Furthermore, large prospective antenatal studies assessing LA muscle passive stiffness before and following vaginal childbirth will be required to investigate this observation further. For instance, we estimate that we would have needed to study a total of 28 women in both groups to detect a 10% difference in pelvic muscle stiffness between women who showed MRI evidence of LA muscle avulsion and those who showed no MRI evidence of avulsion with 80% power. It is also highly likely that other antenatal risk factors, such as primiparity, the size and attitude of the fetal head, ethnicity, maternal age and BMI, would also influence the risk of LA muscle avulsion injuries [7, 23].

Our observation that maternal BMI correlates negatively with PFM stiffness is supported by another report [17], and would also seem consistent with reports of a higher risk of LA muscle injury in women with low BMI [24]. It is plausible that higher PFM stiffness in women with lower BMI could predispose them to LA muscle avulsion, and that raised maternal BMI with greater PFM fat composition could reduce measured stiffness, resulting in more compliance and less risk of muscle avulsion during vaginal childbirth. Further studies are required to clarify these observations.

We have observed that in the postnatal period, the combination of low Oxford Scores, great pelvic muscle stiffness, and lower BMI appear to be strongly predictive of presentation with urinary/bowel/sexual symptoms (predictive AUC 0.84, sensitivity 85% and specificity 79%). Regarding the relative association of the Oxford Pelvic Score (OPS) with features of LA muscle avulsion, we observed a trend towards lower postnatal OPS with increasing duration of the second stage of labour, instrumental forceps delivery and the presence of symptoms of perineal/pelvic floor trauma. These did not attain statistical significance, perhaps because of the highly limited participant numbers in this exploratory pilot study. Nevertheless, these observations would suggest that although PFM stiffness may reflect pelvic floor predisposition to trauma, OPS better reflects the functional state of perineal and pelvic floor musculature at the time of the assessment, hence its clinical use for assessing PFM tone before and after physiotherapy [25]. OPS would therefore be lower in women who have sustained LA muscle injury than in those who have not [19], as we observed in this study and as has recently been reported by others [26–28].

Our data show that women presenting with symptoms in the PTC were more likely to demonstrate MI evidence of LA muscle defects than their asymptomatic counterparts. However, our numbers were too low for any definitive conclusions to be reached regarding the association of MRI evidence of LA muscle defects with labour and delivery characteristics, such as the duration of the second stage and forceps delivery. This was not the primary objective of this preliminary study, but most previous studies have shown that LA avulsion is strongly associated with prolonged duration of the second stage of labour and with forceps delivery [2, 7].

It has long been recognized that neurological (pudendal nerve) damage is a mechanism of pelvic floor trauma and dysfunction following vaginal childbirth. Many studies have shown prolonged pudendal nerve terminal motor latency (PNTML) [29, 30] following vaginal delivery. The extent to which neurological damage contributes to the PFM stiffness values obtained by the vaginal elastometer is unknown and warrants future studies. It is plausible that elastometry, by assessing the passive stiffness of the LA muscle, offers an objective functional score, which, together with quantitative perineometry or qualitative OPS assessment, can better inform clinical care decision-making, either antenatally or postnatally, offering advantages over non-functional imaging studies by ultrasound or MRI.

Our observational study had a number of limitations. First, we could not ascertain the PFM stiffness values of the studied women antenatally and therefore could not determine the influence of antenatal PFM stiffness on the risk of LA muscle trauma. To clarify any causal relationship between pelvic floor muscle stiffness and levator muscle damage, large longitudinal studies, commenced before delivery, in nulliparous women, would be required following on from a recent pilot study [17]. Such a study design will also enable comparisons between women who sustain LA muscle trauma and those who do not. Limited sample sizes are likely to account for the non-significance of our observations for the primiparous women. In this preliminary pilot study, we focused on elastometry in the specific setting of a PTC attended only by women who experienced OASI during their last childbirth. We aimed to generate initial data on the basis of which definitive larger studies on nulliparous women that included control groups were to be designed and carried out. However, despite this limitation, our study is to our knowledge the first to examine vaginal elastometry in the clinical setting of a PTC. Furthermore, retrospective interrogation of maternity database records did not allow us to obtain detailed information regarding management of the perineum during vaginal birth in this cohort.

We have demonstrated that high pelvic stiffness scores were associated with prolonged second stage of labour requiring forceps delivery in the antecedent pregnancy, evidence of LA muscle avulsion defects and presentation with symptoms. Whether high antenatal pelvic stiffness scores, considered together with other risk factors for LA avulsion during vaginal childbirth, could prove a useful tool for predicting risk of pelvic floor trauma to enable care stratification and risk mitigation remains to be determined through prospective antenatal studies. It has been suggested that modification of PFM stiffness properties, such as by mechanically stretching the LA muscles for several weeks prior to delivery, might reduce the incidence of LA trauma during vaginal birth, raising the possibility that such interventions could be underpinned by serial assessment of PFM stiffness by vaginal elastometry to monitor effectiveness and provide new insight into the mechanics of the LA muscle [16]. Whether elastometry proves to be a useful assessment technique in the antenatal or postnatal clinical arena to screen for risk of PFM damage from childbirth, or a measure of improvement following surgical treatment, physiotherapy or other techniques, remains to be determined.

## Abbreviations

BMI: Body mass index
LA: Levator ani
MRI: Magnetic resonance imaging
OASI: Obstetric anal sphincter injury
PFM: Pelvic floor muscle
PTC: Perineal trauma clinic
